# Genetic alteration, RNA expression, and DNA methylation profiling of coronavirus disease 2019 (COVID-19) receptor ACE2 in malignancies: a pan-cancer analysis

**DOI:** 10.1186/s13045-020-00883-5

**Published:** 2020-05-04

**Authors:** Peiwei Chai, Jie Yu, Shengfang Ge, Renbing Jia, Xianqun Fan

**Affiliations:** Department of Ophthalmology, Shanghai Key Laboratory of Orbital Diseases and Ocular Oncology, Ninth People’s Hospital, Shanghai JiaoTong University School of Medicine, Shanghai, 200025 People’s Republic of China

**Keywords:** COVID-19, ACE2, Expression

## Abstract

The novel coronavirus (2019-nCoV) is an emerging causative agent that was first described in late December 2019 and causes a severe respiratory infection in humans. Notably, many of affected patients of COVID-19 were people with malignancies. Moreover, cancer has been identified as an individual risk factor for COVID-19. In addition, the expression of angiotensin converting enzyme 2 (ACE2), the receptor of COVID-19, were aberrantly expressed in many tumors. However, a systematic analysis of ACE2 aberration remained to be elucidated in human cancers. Here, we analyzed genetic alteration, RNA expression, and DNA methylation of ACE2 across over 30 tumors. Notably, overexpression of ACE2 have been observed in including colon adenocarcinoma (COAD), kidney renal papillary cell carcinoma (KIRP), pancreatic adenocarcinoma (PAAD), rectum adenocarcinoma (READ), stomach adenocarcinoma (STAD), and lung adenocarcinoma (LUAD). In addition, hypo DNA methylation of ACE2 has also been identified in most of these ACE2 highly expressed tumors. Conclusively, our study for the first time curated both genetic and epigenetic variations of ACE2 in human malignancies. Notably, because our study is a bioinformatics assay, further functional and clinical validation is warranted.

To the editor,

The novel Coronavirus disease 2019 (COVID-19) is the causative agent of a severe respiratory infection, which is of global public health concern [1, 2]. To date (17 April 2020), COVID-19 has resulted in a total of 2,175,460 laboratory-confirmed human infections, including 146,536 deaths. Moreover, cancer has been already identified as an individual risk factor for COVID-19 [3, 4]. However, a systematic analysis of ACE2 aberration remained uncharacterized in human cancers.

We then curated a pan-cancer analysis of ACE2 in malignancies. In TCGA pan-cancer panel, the most frequent DNA alteration is mutation. Mutations were mainly distributed in UCEC, SKCM, UCS and STAD (Figure S1A). In another pan-cancer panel, the most frequent DNA alteration is amplification. Amplifications of ACE2 were observed in NEPC and PRAD patients (Figure S1B). In addition, ACE2 mutations in malignancies were distributed across all exons of ACE2 without hot spot mutation site (Figure S2AC, Table S1). The most frequent mutation was H195Y/X195_splice (Figure S2B) and X555_splice (Figure S2D). Two most frequent mutations were distributed in the peptidase domain of ACE2, which were predicted to be inactivating mutations.

We next compared ACE2 expression in tumor and its normal control tissue. ACE2 expression was upregulated in six tumors while downregulated in three tumors (Table S2, Fig. 1a). Notably, COAD, KIRP, PAAD, READ, STAD and LUAD presented with increased ACE2 expression (Fig. 1b). Notably, because COVID-19 was mainly transmitted through air-way, we focus on respiratory system tumors. ACE2 was significantly upregulated in LUAD while remained unchanged in lung squamous cell carcinoma (Figure S3). Three tumors, including TGCT, THCA, and KICH, presented with decreased ACE2 expression (Fig. 1c). We then confirmed the relevance of genetic disorders and ACE2 expression. We found that mutations were not relevant to RNA expression (Figure S4A). In addition, we found DNA copy variation were neither statistically relevant to ACE2 expression (Figure S4B). Thus, it is possible that the upregulation of ACE2 expression was not resulted from genetic variation. We then examined the epigenetic disorders of ACE2 in cancers.

**Fig. 1:**
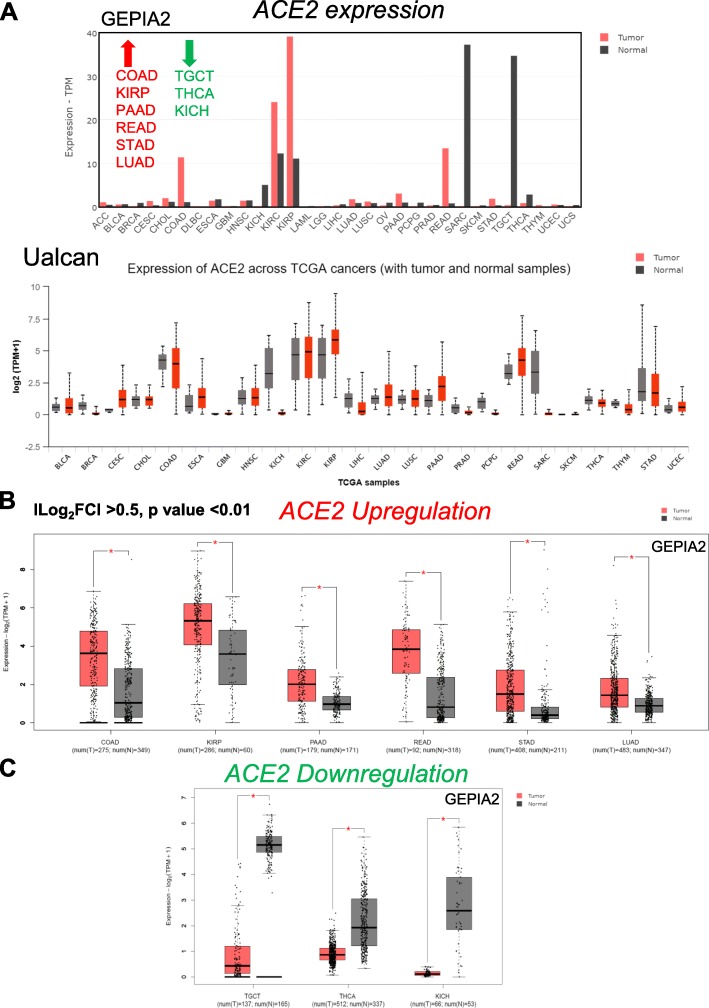
RNA expression aberration of ACE2 in tumors. **a** RNA expression aberration of ACE2 in tumors using GEPIA2 (upper panel) and Ualcan (lower panel) database. Red, tumor samples; grey, normal control samples. **b** Colon adenocarcinoma (COAD), kidney renal papillary cell carcinoma (KIRP), pancreatic adenocarcinoma (PAAD), rectum adenocarcinoma (READ), lung adenocarcinoma (LUAD), and stomach adenocarcinoma (STAD) presented increased ACE2 expression. *p < 0.05. This data was obtained using GEPIA2. Red, tumor samples; grey, normal control samples. **c** Testicular germ cell tumors (TGCT), thyroid carcinoma (THCA), and kidney chromophobe (KICH) presented decreased ACE2 expression. *p < 0.05. This data was obtained using GEPIA2. Red, tumor samples; grey, normal control samples.

Four probes in ACE2 promoter were used for detecting DNA methylation level of ACE2 (Fig. 2a). We have found that the five highly ACE2 express tumors presented with decreased DNA methylation level of ACE2, including COAD, KIRP, PAAD, READ, and LUAD (Fig. 2b). Accordingly, an ACE2 downregulated tumor, TGCT, presented increased DNA methylation level (Fig. 2c). Since there is no available DNA methylation dataset for normal control of KICH, we could not compare global DNA methylation level of KICH. Instead, we compared DNA methylation level of KICH across different tumor stages, and we found that the DNA methylation was not significantly changed (Figure S5). In addition, DNA methylation level of ACE2 in THCA and STAD remained unchanged (Figure S6AB), which suggested DNA methylation might be not the only reason of abnormal ACE2 expression. Other possibilities, such as histone modifications [5] and glycosylation [6] may give rise in the abnormal expression of ACE2, which requires further explorations.

**Fig. 2:**
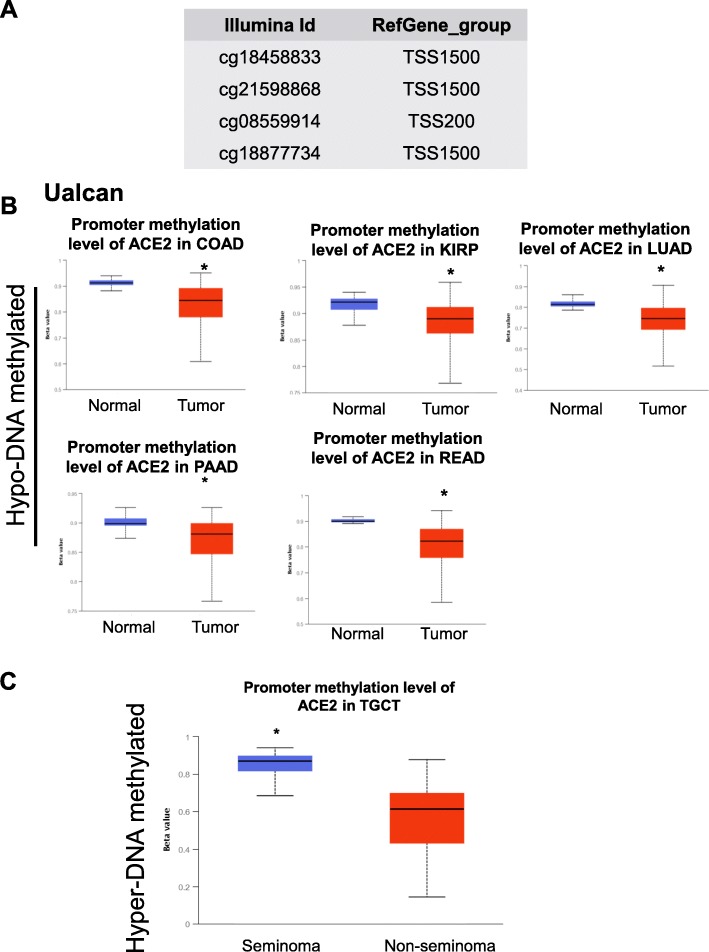
DNA methylation aberration of ACE2 in tumors. **a** Probes for detecting DNA methylation of ACE2 promoter. **b** Four highly ACE2 express tumors presented with decreased DNA methylation level of ACE2, including colon adenocarcinoma (COAD), kidney renal papillary cell carcinoma (KIRP), pancreatic adenocarcinoma (PAAD), and rectum adenocarcinoma (READ). *p < 0.05. This data was obtained using Ualcan. **c** An ACE2 downregulated tumor, testicular germ cell tumors (TGCT), presented increased DNA methylation level. *p < 0.05. This data was obtained using Ualcan.

We then explored the impact of ACE2 expression in overall survival (OS) and disease-free survival (DFS). Six tumors (COAD, KIRP, PAAD, READ, STAD, and LUAD) presented with elevated ACE2 expression while three other tumors (TGCT, THCA, KICH) presented with decreased ACE2 expression; however, ACE2 expression was not statistically relevant to patients’ prognosis, neither in DFS (Figure S7) nor OS (Figure S8). For DFS, higher ACE2 expression predicted better outcome in KIRC, LIHC, LUSC, UCS, and OV groups (Figure S9A). For overall survival, higher ACE2 expression indicated better prognosis in KIRC, LIHC, and OV groups. However, higher ACE2 expression in LGG refers to unfavorable outcome, which indicated the ACE2 might function as a dual-edged sword for patients’ outcome (Figure S9B). Notably, ACE2 has been proven to be an important regulator in tumorigenesis. For instance, ACE2 inhibits breast cancer angiogenesis through suppressing VEGFa/VEGFR2/ERK pathway [7] and reduces cell invasion and migration in NSCLC cells [8].

Among these COVIDs with malignancies, LUAD was the most frequent type [3]. Moreover, patients with lung cancer were confirmed to harbor a higher incidence of COVID-19, with severer symptoms [3, 4]. Here, we have also proved that the ACE2 RNA expression was upregulated in LUADs. Since our study is only a database analysis, further validation in larger clinical cohort is warranted.

## Supplementary information

**Additional file 1: Supplementary Figures. Figure S1:** Genetic aberration of ACE2 in tumors. (**A**) Genetic aberration of ACE2 in tumors using cBioPortal –TCGA pan-cancer panel. This data includes 10953 patients / 10967 samples in 32 studies. The global genetic alteration frequency is 2.3%. (**B**) Genetic aberration of ACE2 in tumors using cBioPortal –Mixed pan-cancer panel. This data includes 21380 patients / 23059 samples in 131 studies. Tumors without any alterations were not listed. The global genetic alteration frequency is 1.2%. **Figure S2:** Mutations in ACE2. (**A**) ACE2 mutations were distributed across all exons of ACE2 without hot spot mutation site in TCGA cohort using cBioPortal. (**B**) The most frequent mutation was H195Y / X195_splice (label in yellow) in TCGA cohort. (**C**) ACE2 mutations were distributed across all exons of ACE2 without hot spot mutation site in Mixed pan-cancer cohort using cBioPortal. (**D**) The most frequent mutation was X555_splice (label in yellow) in Mixed pan-cancer cohort. **Figure S3:** ACE2 expression remained unchanged in lung squamous cell carcinoma (LUSC). **Figure S4:** The relevance of genetic disorders and ACE2 expression. (A) mutations were not relevant to RNA expression. (B) DNA copy variation were neither statistically relevant to RNA ACE2 expression in most cases. **Figure S5:** The DNA methylation level of different stages of KICH. **Figure S6:** The DNA methylation level of thyroid carcinoma (THCA) and stomach adenocarcinoma (STAD). DNA methylation level of ACE2 in THCA (**A**) and STAD (**B**) remained unchanged. **Figure S7:** Disease free survival (DFS) data in ACE2 abnormally expressed malignancies. (A) Survival map in ACE2-abormally expressed tumors; (B) DFS in ACE2 overexpressed tumors, logrank p>0.05; (C) DFS in ACE2 decreased tumors, logrank p> 0.05. **Figure S8:** Overall survival (OS) data in ACE2 abnormally expressed malignancies. (A) Survival map in ACE2-abormally expressed tumors; (B) OS in ACE2 overexpressed tumors, logrank p>0.05; (C) OS in ACE2 decreased tumors, logrank *p*> 0.05. **Figure S9:** Survival map analysis of ACE2 expression. (**A**) Disease free survival map analysis of ACE2 expression. Higher ACE2 expression predicted better outcome, including KICH and kidney renal clear cell carcinoma (KIRC), liver hepatocellular carcinoma (LIHC), lung squamous cell carcinoma (LUSC), uterine carcinosarcoma (UCS) and ovarian serous cystadenocarcinoma (OV) groups. (**B**) Overall survival map analysis of ACE2 expression. Higher ACE2 expression indicated better prognosis in KIRC, LIHC and OV groups. However, higher ACE2 expression in brain lower grade glioma (LGG) refer to unfavorable outcome

**Additional file 2: Table S1.** Mutation spectrum of ACE2 across tumor samples.

**Additional file 3: Table S2.** The TPM expression of ACE2 in 30 kinds of tumors from TCGA database.

**Additional file 4: Table S3.** Dataset sources used in the study.

## Data Availability

Patients data were acquired from cBioportal (https://www.cbioportal.org/), UALCAN (http://ualcan.path.uab.edu/), and gepia2 (http://gepia2.cancer-pku.cn) database tool. We have referred DNA alterations, expression profiling, DNA methylation level of ACE2 promoter, mutation analysis, and Kaplan merrier analysis section (Table S3).

## Abbreviations

COVID-19: Novel coronavirus disease 2019
ACE2: Angiotensin converting enzyme 2
TCGA: The cancer genome atlas
UCEC: Uterine corpus endometrial carcinoma
SKCM: Skin cutaneous melanoma
THCA: Thyroid carcinoma
COAD: Colon adenocarcinoma
KIRP: Kidney renal papillary cell carcinoma
PAAD: Pancreatic adenocarcinoma
READ: Rectum adenocarcinoma
STAD: Stomach adenocarcinoma
NEPC: Neuroendocrine prostate cancers
UCS: Uterine carcinosarcoma
PRAD: Prostate adenocarcinoma
KIRC: Kidney renal clear cell carcinoma
KIRP: Kidney renal papillary cell carcinoma
KICH: Kidney chromophobe
LIHC: Liver hepatocellular carcinoma
LUSC: Lung squamous cell carcinoma
OV: Ovarian serous cystadenocarcinoma
LUAD: Lung adenocarcinoma
NSCLC: Non-small cell lung cancer
OS: Overall survival
DFS: Disease-free survival
LGG: Brain lower-grade glioma
