# Supplementary material for: Genetic alteration, RNA expression, and DNA methylation profiling of coronavirus disease 2019 (COVID-19) receptor ACE2 in malignancies: a pan-cancer analysis
Source: J Hematol Oncol. 2020 May 4;13:43. doi: 10.1186/s13045-020-00883-5 (PMC7197362; doi:10.1186/s13045-020-00883-5)

Figure S1

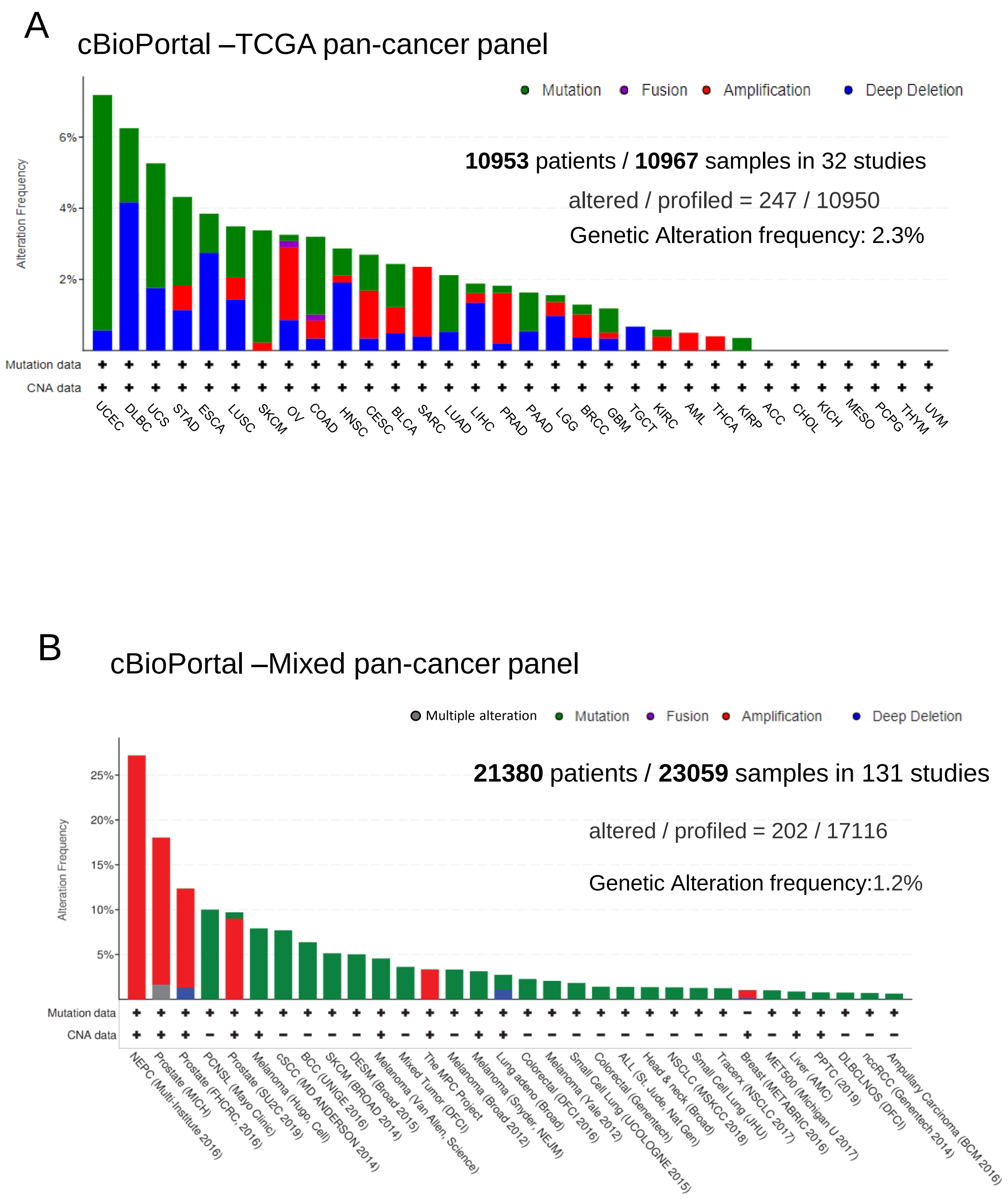
A
cBioPortal –TCGA pan-cancer panel
Mutation
Fusion
Amplification
Deep Deletion
10953 patients / 10967 samples in 32 studies
altered / profiled = 247 / 10950
Genetic Alteration frequency: 2.3%
Alteration Frequency
6%
4%
2%
Mutation data
CNA data
UCEC DLBC UCS STAD ESCA LUSC SKCM OV COAD HNSC CESC BLCA SARC LUAD LIHC PRAD PAAD LGG BRCC GBM TGCT KIRC AML THCA KIRP ACC CHOL KICH MESO PCPG THYM UVM
B
cBioPortal –Mixed pan-cancer panel
Multiple alteration
Mutation
Fusion
Amplification
Deep Deletion
21380 patients / 23059 samples in 131 studies
altered / profiled = 202 / 17116
Genetic Alteration frequency:1.2%
Alteration Frequency
25%
20%
15%
10%
5%
Mutation data
CNA data
NEPC (Multi-Institute 2016)
Prostate (MICH)
Prostate (FHCRC 2016)
PCNSL (Mayo Clinic)
Prostate (SU2C 2019)
Melanoma (Hugo, Cell)
cSCC (MD ANDERSON 2014)
BCC (UNIGE 2016)
SKCM (BROAD 2014)
DESM (Broad 2015)
Melanoma (Van Allen, Science)
Mixed Tumor (DFCI)
The MPC Project
Melanoma (Broad 2012)
Lung adeno (Snyder, NEJM)
Colorectal (Broad)
Melanoma (DFCI 2016)
Small Cell Lung (Yale 2012)
Colorectal (UCOLOGNE 2015)
ALL (St. Jude, Nat Gen)
Head & neck (Broad)
NSCLC (MSKCC 2018)
Small Cell Lung (JHU)
Tracerx (NSCLC 2017)
Breast (METABRIC 2016)
MET500 (Michigan U 2017)
Liver (AMC)
PPTC (2019)
DLBCLNOS (DFCI)
nccRCC (Genentech 2014)
Ampullary Carcinoma (BCM 2016)

Figure S2

A

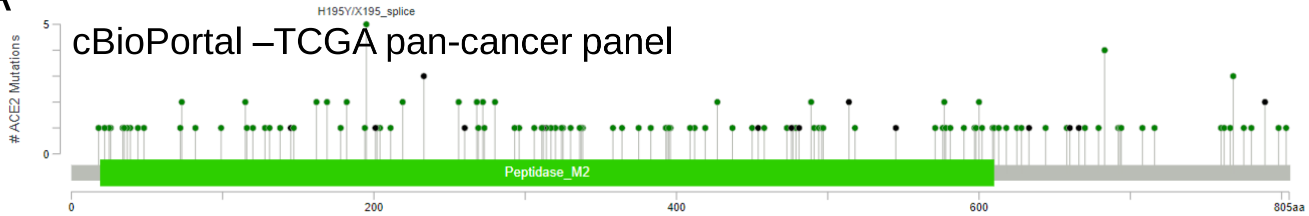

H195Y / X195_splice

Study	Sample ID	Cancer Type	Protein Change	Annotation ▼	Mutation Type	Copy #	COSMIC	Allele Freq (T)	# Mut in Sample
Colorectal Adeno...	TCGA-AG-A002-01	Rectal Adenocarcinoma	*H195Y*	○	Missense	Diploid	2	0.84	11438
Skin Cutaneous M...	TCGA-EE-A29N-06	Cutaneous Melanoma	*H195Y*	○	Missense	Diploid	2	0.39	575
Uterine Corpus E...	TCGA-AP-A1E0-01	Uterine Endometrioid Carcinoma	*H195Y*	○	Missense	Diploid	2	0.61	4834
Lung Squamous Ce...	TCGA-L3-A524-01	Lung Squamous Cell Carcinoma	*X195_splice*	○	Splice	Diploid		0.30	299
Liver Hepatocell...	TCGA-DD-AAW2-01	Hepatocellular Carcinoma	*X195_splice*	○	Splice	Gain		0.90	116

B

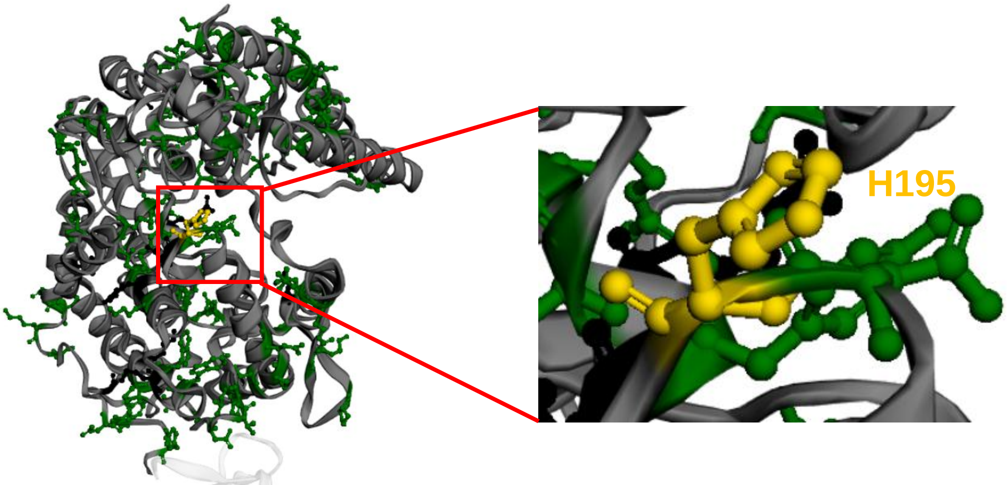

C

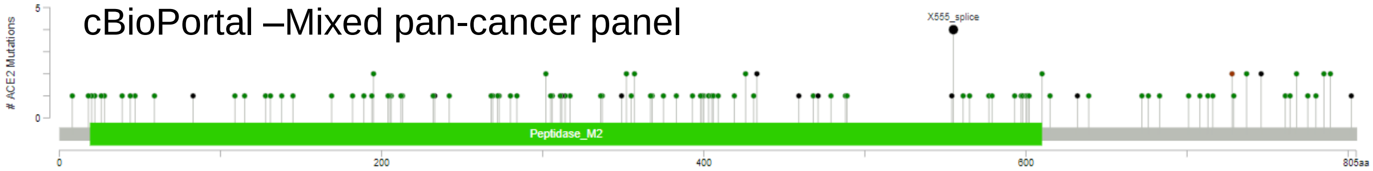

X555_splice

Study	Sample ID	Cancer Type	Protein Change	Annotation ▼	Mutation Type	Copy #	COSMIC	Allele Freq (T)	# Mut in Sample
Colorectal Adeno...	coadread_dfci_20...	Colorectal Adenocarcinoma	*X555_splice*	○	Splice				1481
Colorectal Adeno...	coadread_dfci_20...	Colorectal Adenocarcinoma	*X555_splice*	○	Splice				72
Pancreatic Adeno...	ICGC_0548	Pancreatic Adenocarcinoma	*X555_splice*	○	Splice				984
Basal Cell Carci...	5-PT035-T1	Skin Cancer, Non-Melanoma	*X555_splice*	○	Splice			0.27	2823

Showing 1-4 of 4 Mutations

D

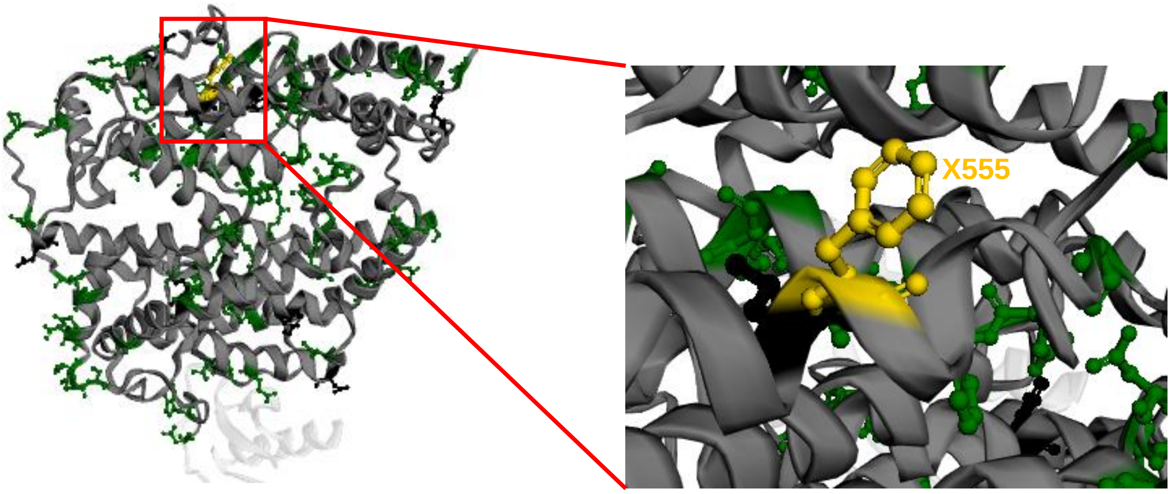

Figure S3

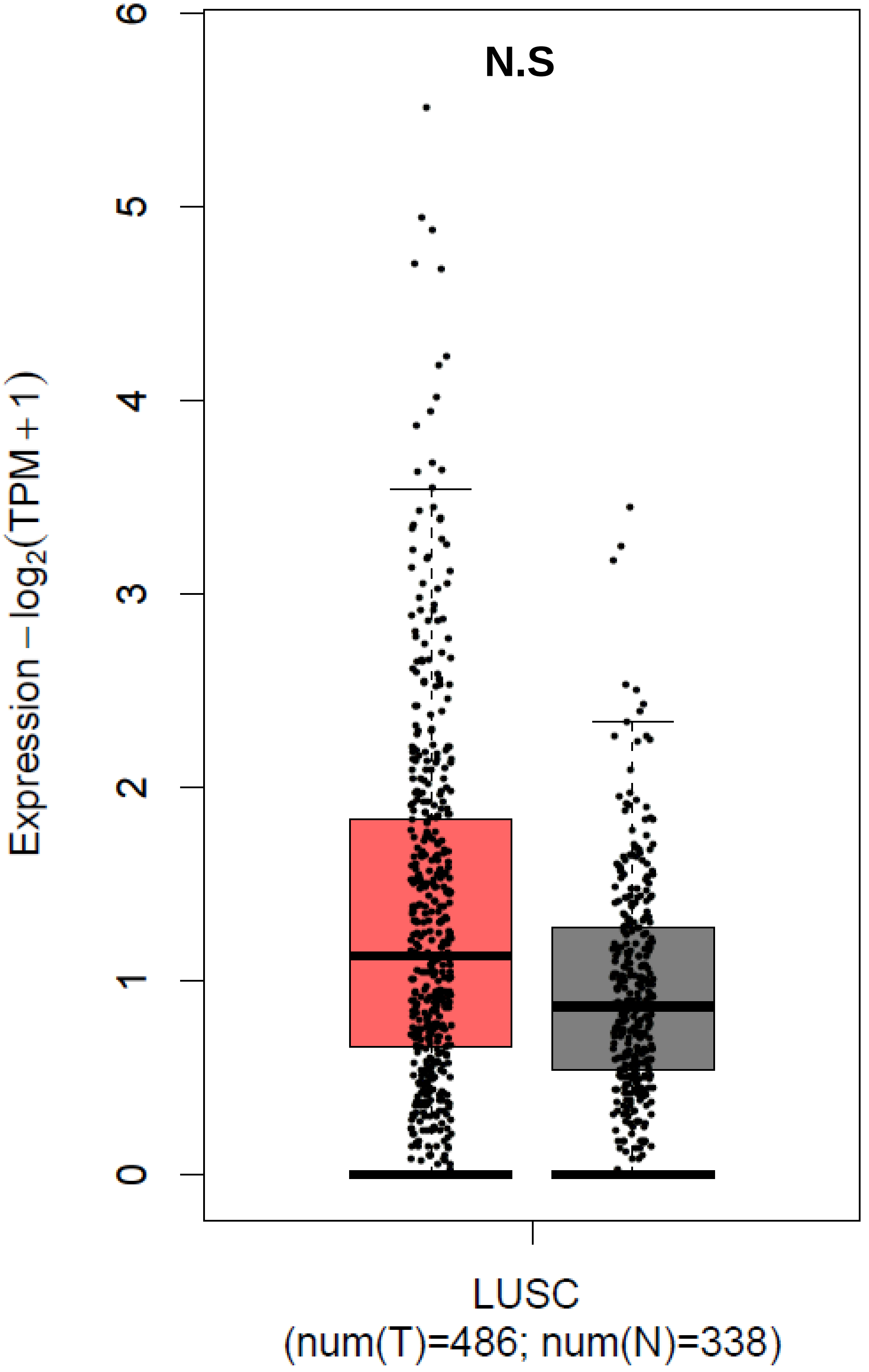

Figure S4

A

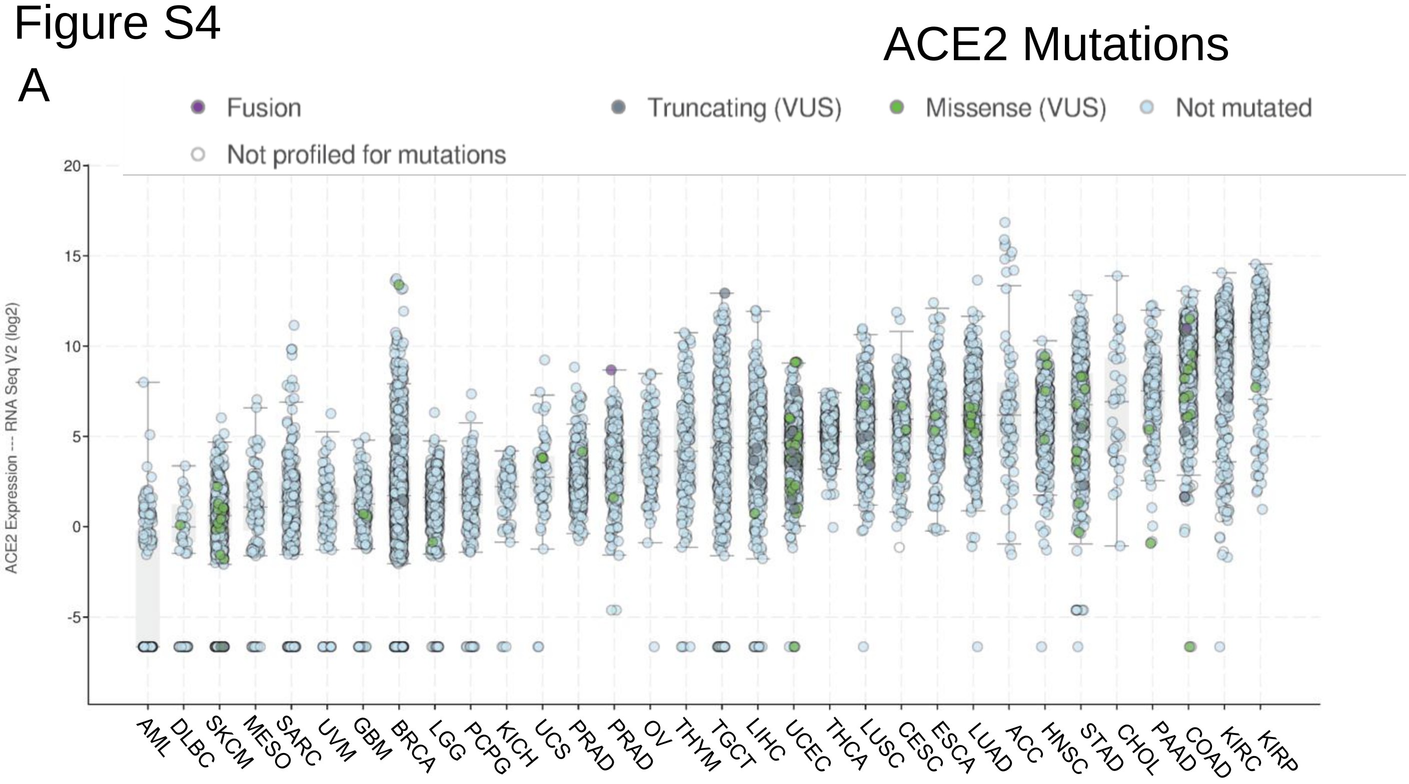
ACE2 Mutations
Fusion
Truncating (VUS)
Missense (VUS)
Not mutated
Not profiled for mutations
ACE2 Expression --- RNA Seq V2 (log2)
20
15
10
5
0
-5
AML
DLBC
SKCM
MESO
SARC
UVM
GBM
BRCA
LGG
PCPG
KICH
UCS
PRAD
PRAD
OV
THYM
TGCT
LIHC
UCEC
THCA
LUSC
CESC
ESCA
LUAD
ACC
HNSC
STAD
CHOL
PAAD
COAD
KIRC
KIRP

B

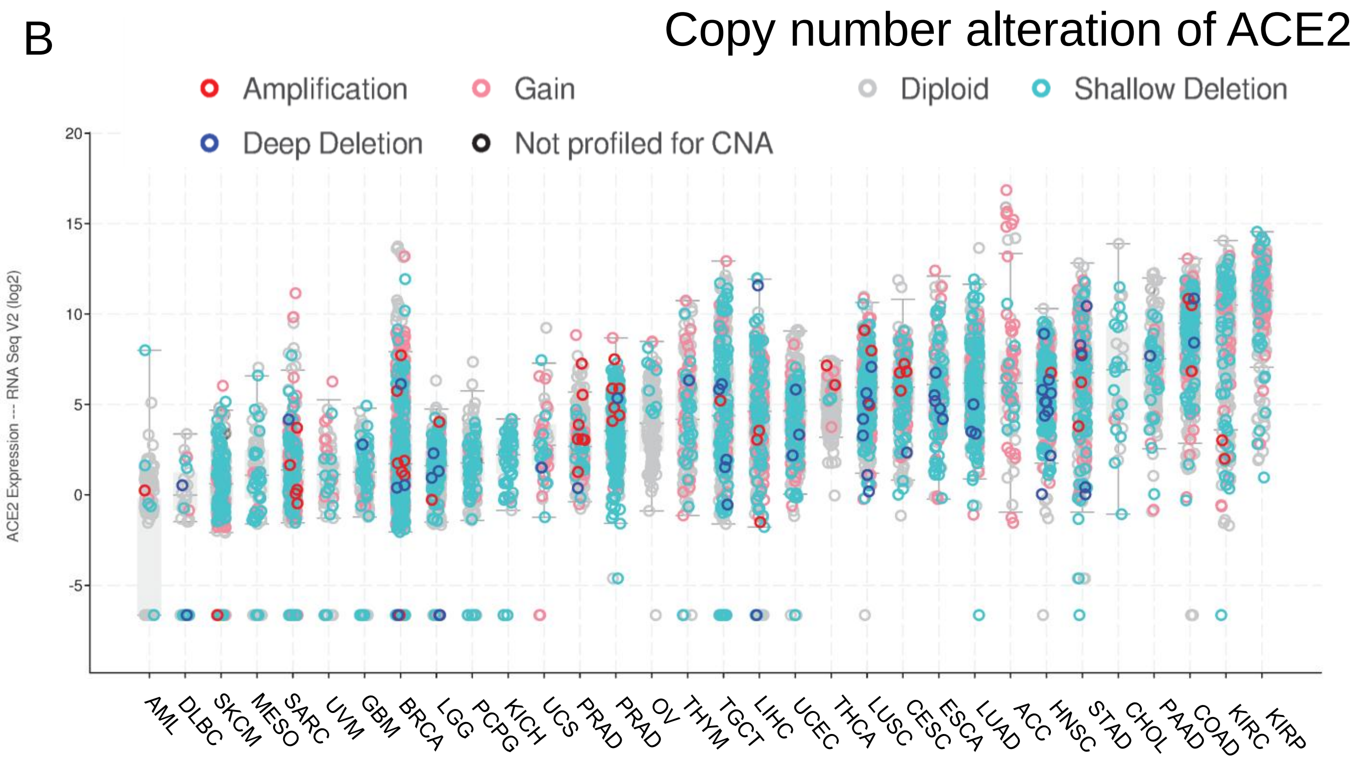
Copy number alteration of ACE2
Amplification
Gain
Diploid
Shallow Deletion
Deep Deletion
Not profiled for CNA
ACE2 Expression --- RNA Seq V2 (log2)
20
15
10
5
0
-5
AML
DLBC
SKCM
MESO
SARC
UVM
GBM
BRCA
LGG
PCPG
KICH
UCS
PRAD
PRAD
OV
THYM
TGCT
LIHC
UCEC
THCA
LUSC
CESC
ESCA
LUAD
ACC
HNSC
STAD
CHOL
PAAD
COAD
KIRC
KIRP

Figure S5

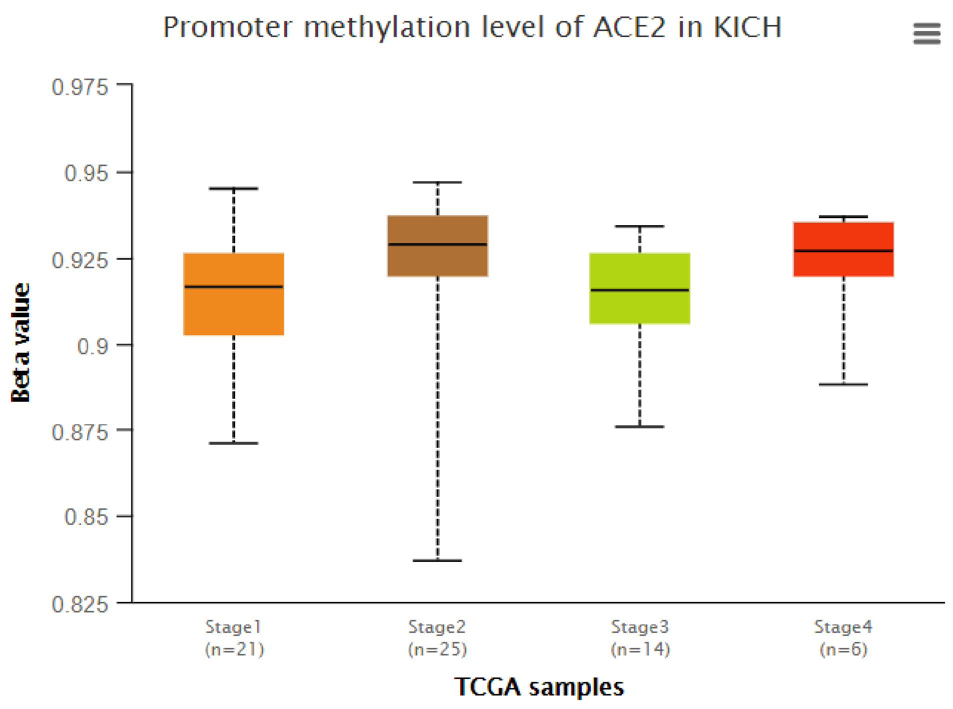

Promoter methylation level of ACE2 in KICH
Beta value
0.975
0.95
0.925
0.9
0.875
0.85
0.825
Stage1
(n=21)
Stage2
(n=25)
Stage3
(n=14)
Stage4
(n=6)
TCGA samples

Figure S6

A

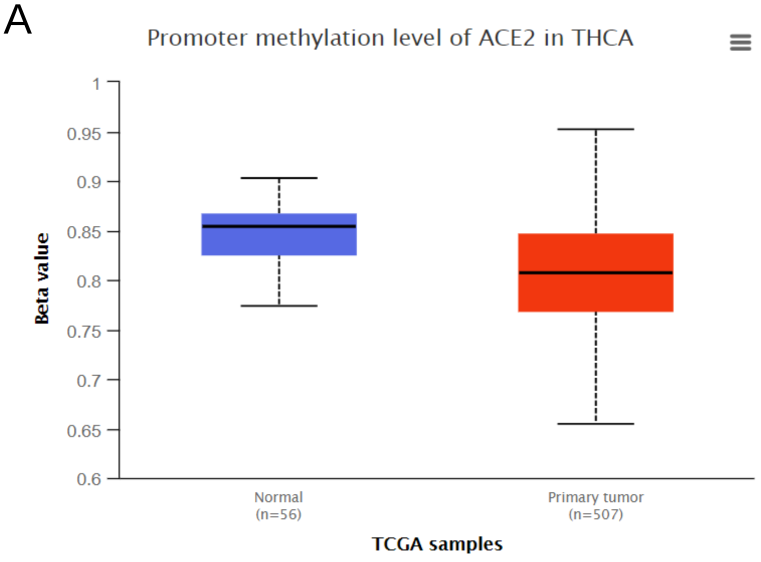

Promoter methylation level of ACE2 in THCA
Beta value
1
0.95
0.9
0.85
0.8
0.75
0.7
0.65
0.6
Normal
(n=56)
Primary tumor
(n=507)
TCGA samples

B

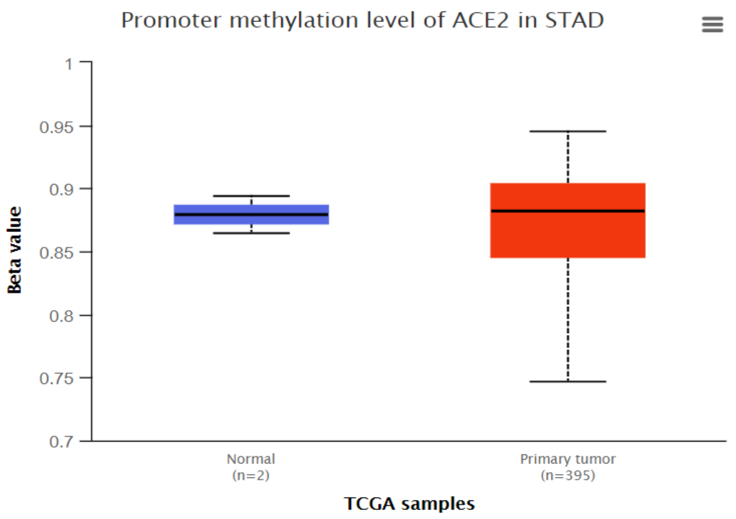

Promoter methylation level of ACE2 in STAD
Beta value
1
0.95
0.9
0.85
0.8
0.75
0.7
Normal
(n=2)
Primary tumor
(n=395)
TCGA samples

Figure S7

Disease free survival

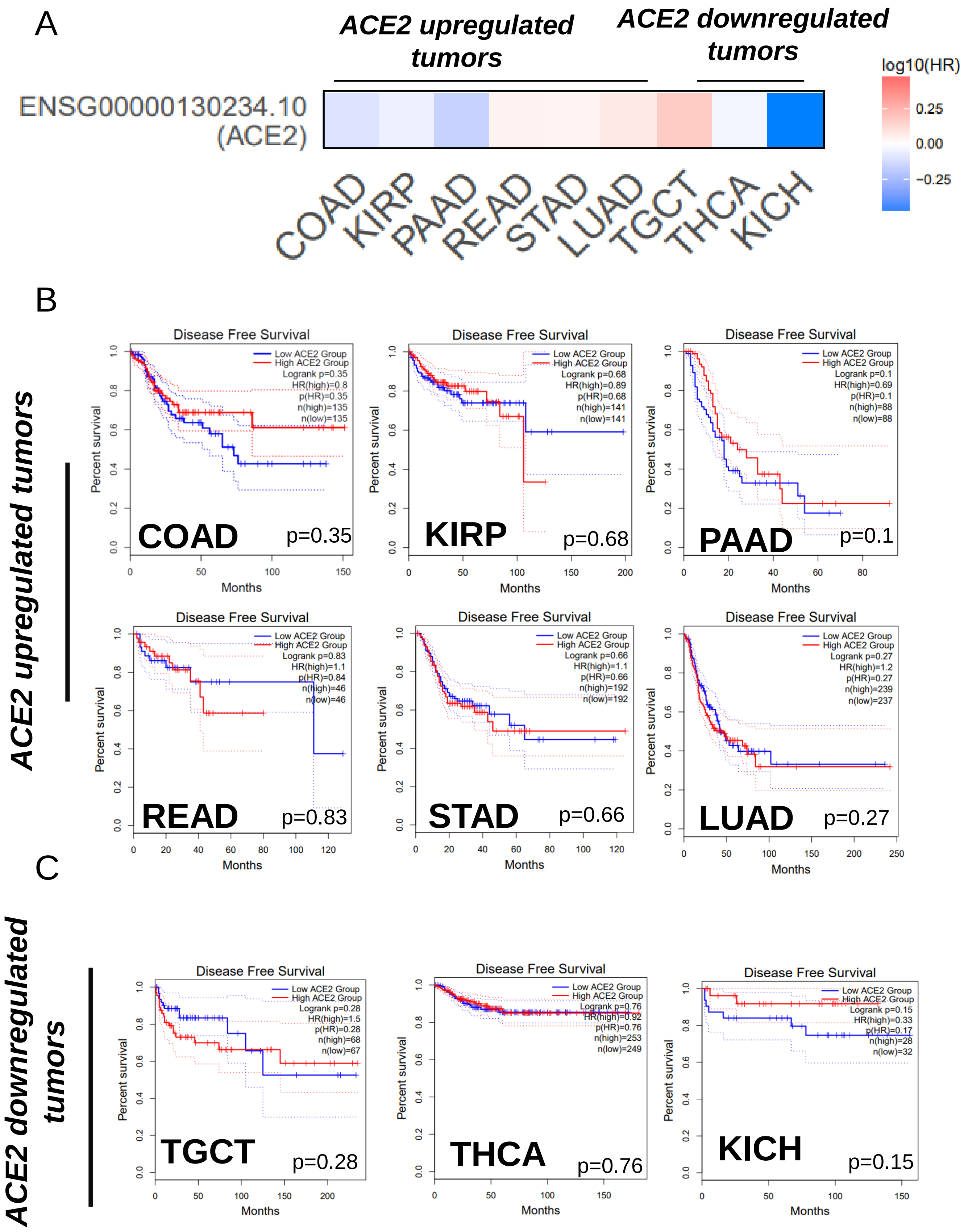

Figure S8

Overall survival

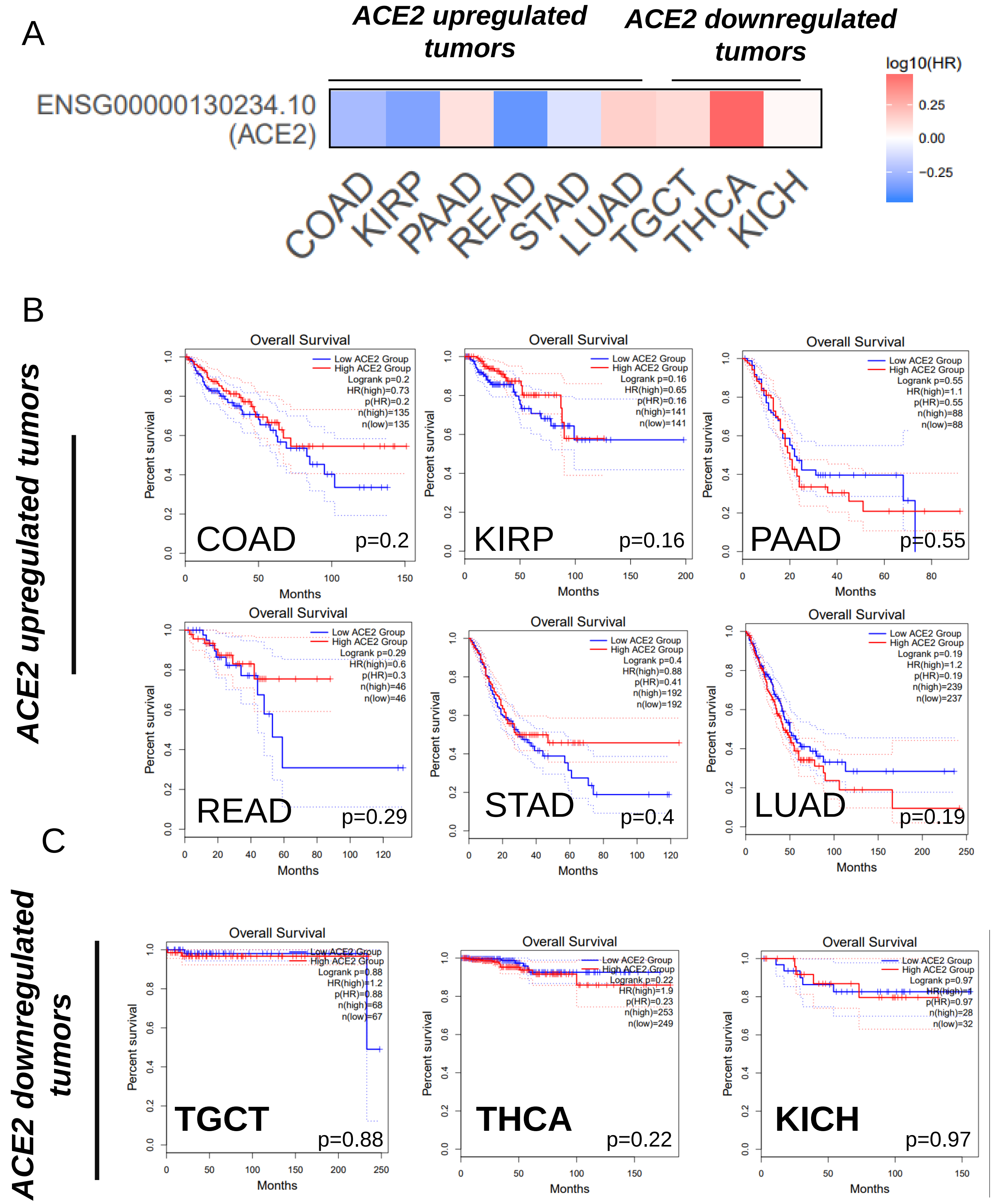

Figure S9

A

Disease free survival

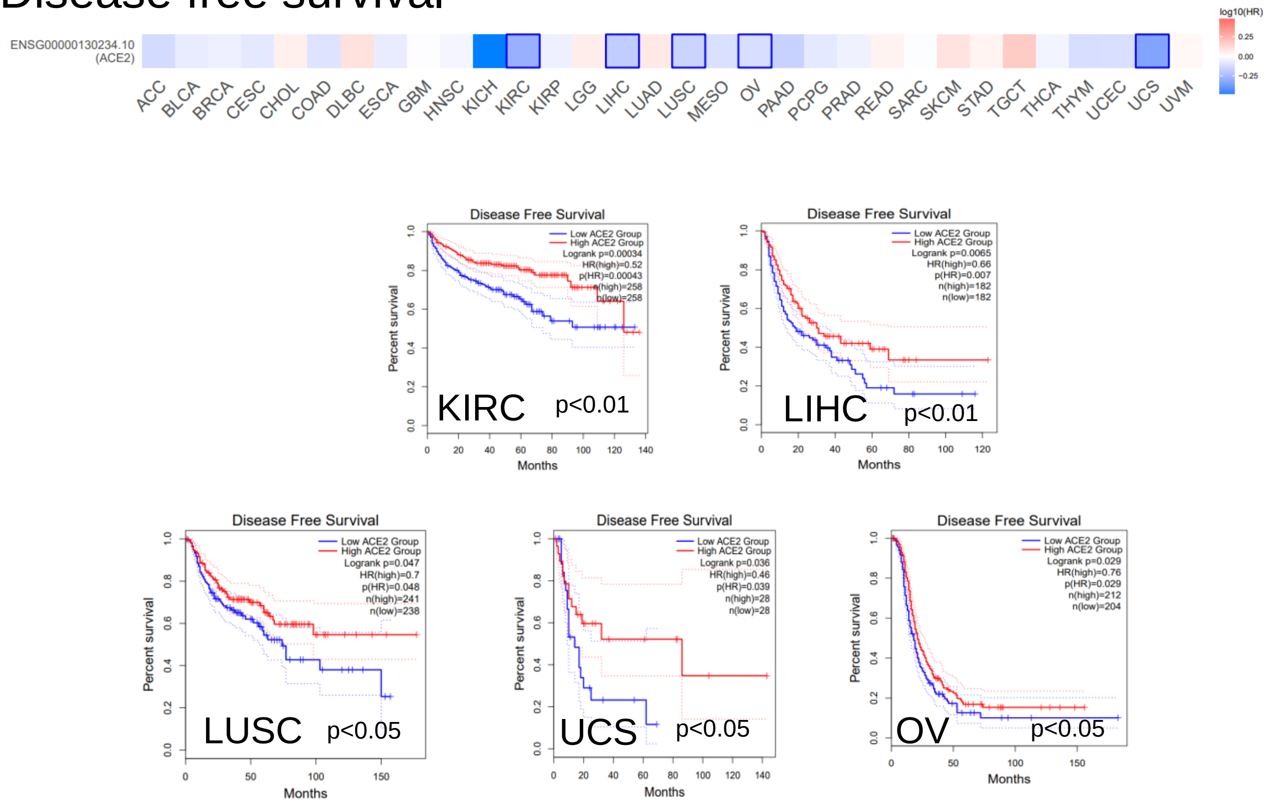

B

Overall survival

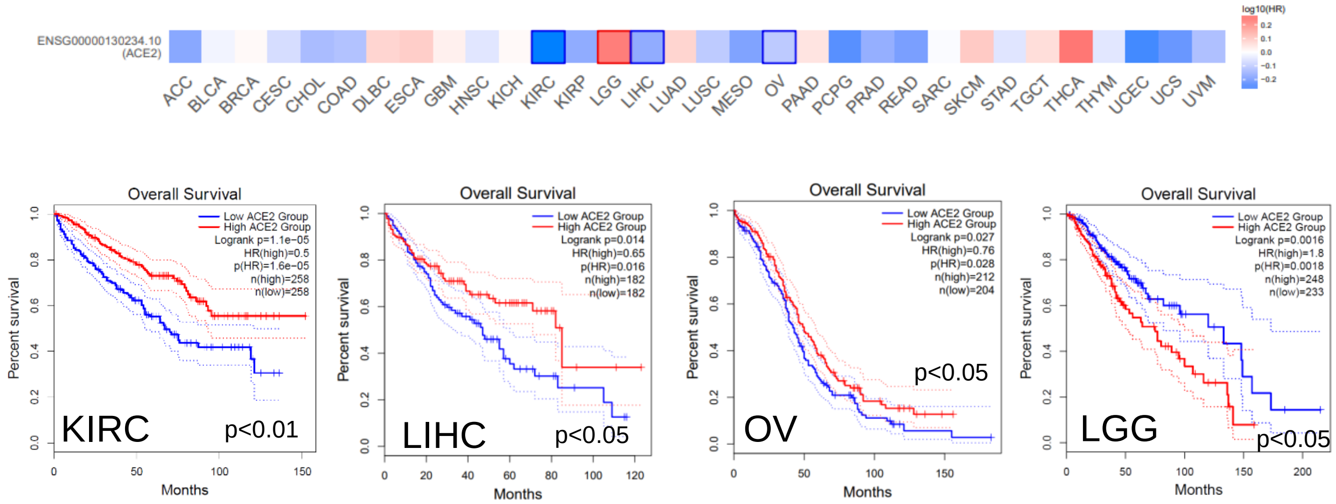

Supplement: Supplementary file 1 — Additional file 1: Supplementary Figures. Figure S1: Genetic aberration of ACE2 in tumors. (A) Genetic aberration of ACE2 in tumors using cBioPortal –TCGA pan-cancer panel. This data includes 10953 patients / 10967 samples in 32 studies. The global genetic alteration frequency is 2.3%. (B) Genetic aberration of ACE2 in tumors using cBioPortal –Mixed pan-cancer panel. This data includes 21380 patients / 23059 samples in 131 studies. Tumors without any alterations were not listed. The global genetic alteration frequency is 1.2%. Figure S2: Mutations in ACE2. (A) ACE2 mutations were distributed across all exons of ACE2 without hot spot mutation site in TCGA cohort using cBioPortal. (B) The most frequent mutation was H195Y / X195_splice (label in yellow) in TCGA cohort. (C) ACE2 mutations were distributed across all exons of ACE2 without hot spot mutation site in Mixed pan-cancer cohort using cBioPortal. (D) The most frequent mutation was X555_splice (label in yellow) in Mixed pan-cancer cohort. Figure S3: ACE2 expression remained unchanged in lung squamous cell carcinoma (LUSC). Figure S4: The relevance of genetic disorders and ACE2 expression. (A) mutations were not relevant to RNA expression. (B) DNA copy variation were neither statistically relevant to RNA ACE2 expression in most cases. Figure S5: The DNA methylation level of different stages of KICH. Figure S6: The DNA methylation level of thyroid carcinoma (THCA) and stomach adenocarcinoma (STAD). DNA methylation level of ACE2 in THCA (A) and STAD (B) remained unchanged. Figure S7: Disease free survival (DFS) data in ACE2 abnormally expressed malignancies. (A) Survival map in ACE2-abormally expressed tumors; (B) DFS in ACE2 overexpressed tumors, logrank p>0.05; (C) DFS in ACE2 decreased tumors, logrank p> 0.05. Figure S8: Overall survival (OS) data in ACE2 abnormally expressed malignancies. (A) Survival map in ACE2-abormally expressed tumors; (B) OS in ACE2 overexpressed tumors, logrank p>0.05; (C) OS in ACE2 decr [file 13045_2020_883_MOESM1_ESM.pdf]
