# Supplementary material for: Genetic alteration, RNA expression, and DNA methylation profiling of coronavirus disease 2019 (COVID-19) receptor ACE2 in malignancies: a pan-cancer analysis
Source: J Hematol Oncol. 2020 May 4;13:43. doi: 10.1186/s13045-020-00883-5 (PMC7197362; doi:10.1186/s13045-020-00883-5)
Supplement: Supplementary file 2 — Additional file 2: Table S1. Mutation spectrum of ACE2 across tumor samples. [file 13045_2020_883_MOESM2_ESM.docx]

Table S1 Mutation spectrum of ACE2 across tumor samples.

| **Sample ID** | **Cancer Type** | **Protein Change** | **Mutation Type** | **Copy #** | **Allele Freq (T)** |
| --- | --- | --- | --- | --- | --- |
| TCGA-E2-A15L-01 | Breast Invasive Lobular Carcinoma | ***E145**** | **Nonsense** | Diploid | 0.49 |
| TCGA-06-6389-01 | Glioblastoma Multiforme | ***V581I*** | **Missense** | Diploid | 0.25 |
| TCGA-19-1390-01 | Glioblastoma Multiforme | ***R766K*** | **Missense** | **ShallowDel** | 0.25 |
| TCGA-95-7948-01 | Lung Adenocarcinoma | ***I256M*** | **Missense** | Diploid | 0.05 |
| TCGA-HC-A76W-01 | Prostate Adenocarcinoma | ***I256M*** | **Missense** | Diploid | 0.61 |
| TCGA-BR-4201-01 | Stomach Adenocarcinoma | ***R644Q*** | **Missense** | Diploid | 0.15 |
| TCGA-BR-4292-01 | Stomach Adenocarcinoma | ***R219H*** | **Missense** | Diploid | 0.26 |
| TCGA-BR-7707-01 | Stomach Adenocarcinoma | ***A412T*** | **Missense** | Diploid | 0.07 |
| TCGA-D7-6527-01 | Papillary Stomach Adenocarcinoma | ***K458T*** | **Missense** | **ShallowDel** | 0.56 |
| TCGA-D7-6528-01 | Tubular Stomach Adenocarcinoma | ***N330H*** | **Missense** | **ShallowDel** | 0.12 |
| TCGA-F1-6874-01 | Intestinal Type Stomach Adenocarcinoma | ***P590L*** | **Missense** | Diploid | 0.69 |
| TCGA-HU-A4GN-01 | Tubular Stomach Adenocarcinoma | ***L73**** | **FS del** | Diploid | 0.12 |
| TCGA-AG-A002-01 | Rectal Adenocarcinoma | ***R169I*** | **Missense** | Diploid | 0.83 |
| TCGA-B5-A11E-01 | Uterine Endometrioid Carcinoma | ***R169I*** | **Missense** | Diploid | 0.42 |
| TCGA-CR-6484-01 | Head and Neck Squamous Cell Ca... | ***I694M*** | **Missense** | Diploid | 0.17 |
| TCGA-44-2659-01 | Lung Adenocarcinoma | ***R219P*** | **Missense** | **ShallowDel** | 0.2 |
| TCGA-44-7670-01 | Lung Adenocarcinoma | ***G211W*** | **Missense** | **Gain** | 0.28 |
| TCGA-69-7980-01 | Lung Adenocarcinoma | ***L320F*** | **Missense** | **Gain** | 0.22 |
| TCGA-73-4658-01 | Lung Adenocarcinoma | ***D693N*** | **Missense** | Diploid | 0.08 |
| TCGA-99-7458-01 | Lung Adenocarcinoma | ***H34N*** | **Missense** | Diploid | 0.13 |
| TCGA-AP-A056-01 | Uterine Endometrioid Carcinoma | ***E479D*** | **Missense** | Diploid | 0.3 |
| TCGA-AP-A056-01 | Uterine Endometrioid Carcinoma | ***R306I*** | **Missense** | Diploid | 0.32 |
| TCGA-AP-A056-01 | Uterine Endometrioid Carcinoma | ***N194K*** | **Missense** | Diploid | 0.27 |
| TCGA-BT-A2LD-01 | Bladder Urothelial Carcinoma | ***X233_splice*** | **Splice** | Diploid | 0.45 |
| TCGA-AP-A059-01 | Uterine Endometrioid Carcinoma | ***X233_splice*** | **Splice** | Diploid | 0.13 |
| TCGA-AP-A059-01 | Uterine Endometrioid Carcinoma | ***R204I*** | **Missense** | Diploid | 0.15 |
| TCGA-AP-A0LM-01 | Uterine Endometrioid Carcinoma | ***L39M*** | **Missense** | Diploid | 0.24 |
| TCGA-AG-3892-01 | Rectal Adenocarcinoma | ***F683L*** | **Missense** | Diploid | 0.04 |
| TCGA-F5-6814-01 | Rectal Adenocarcinoma | ***F683L*** | **Missense** | Diploid | 0.52 |
| TCGA-AP-A1E0-01 | Uterine Endometrioid Carcinoma | ***F683L*** | **Missense** | Diploid | 0.44 |
| TCGA-AX-A0J0-01 | Uterine Endometrioid Carcinoma | ***F683L*** | **Missense** | Diploid | 0.44 |
| TCGA-AX-A0J0-01 | Uterine Endometrioid Carcinoma | ***K131Q*** | **Missense** | Diploid | 0.42 |
| TCGA-B5-A11E-01 | Uterine Endometrioid Carcinoma | ***S602Y*** | **Missense** | Diploid | 0.37 |
| TCGA-B5-A11E-01 | Uterine Endometrioid Carcinoma | ***S128I*** | **Missense** | Diploid | 0.4 |
| TCGA-B5-A11H-01 | Uterine Endometrioid Carcinoma | ***P336S*** | **Missense** | Diploid | 0.23 |
| TCGA-B5-A11N-01 | Uterine Endometrioid Carcinoma | ***K26N*** | **Missense** | Diploid | 0.26 |
| TCGA-BS-A0UF-01 | Uterine Endometrioid Carcinoma | ***K419T*** | **Missense** | Diploid | 0.32 |
| TCGA-BS-A0UJ-01 | Uterine Endometrioid Carcinoma | ***R775I*** | **Missense** | Diploid | 0.41 |
| TCGA-A5-A0G2-01 | Uterine Serous Carcinoma/Uteri... | ***G789**** | **Nonsense** | Diploid | 0.72 |
| TCGA-BS-A0UV-01 | Uterine Endometrioid Carcinoma | ***G789**** | **Nonsense** | Diploid | 0.38 |
| TCGA-A5-A2K5-01 | Uterine Endometrioid Carcinoma | ***K600N*** | **Missense** | Diploid | 0.21 |
| TCGA-BS-A0UV-01 | Uterine Endometrioid Carcinoma | ***K600N*** | **Missense** | Diploid | 0.42 |
| TCGA-BS-A0UV-01 | Uterine Endometrioid Carcinoma | ***F314L*** | **Missense** | Diploid | 0.41 |
| TCGA-D1-A103-01 | Uterine Endometrioid Carcinoma | ***R273K*** | **Missense** | Diploid | 0.33 |
| TCGA-D1-A17Q-01 | Uterine Endometrioid Carcinoma | ***R768W*** | **Missense** | Diploid | 0.46 |
| TCGA-E6-A1LX-01 | Uterine Endometrioid Carcinoma | ***R768W*** | **Missense** | Diploid | 0.16 |
| TCGA-EO-A22U-01 | Uterine Endometrioid Carcinoma | ***R768W*** | **Missense** | Diploid | 0.43 |
| TCGA-A5-A0G2-01 | Uterine Serous Carcinoma/Uteri... | ***K577N*** | **Missense** | Diploid | 0.71 |
| TCGA-D1-A17Q-01 | Uterine Endometrioid Carcinoma | ***K577N*** | **Missense** | Diploid | 0.27 |
| TCGA-D1-A17Q-01 | Uterine Endometrioid Carcinoma | ***E375D*** | **Missense** | Diploid | 0.43 |
| TCGA-CU-A0YR-01 | Bladder Urothelial Carcinoma | ***Q325P*** | **Missense** | Diploid | 0.59 |
| TCGA-B0-5709-01 | Renal Clear Cell Carcinoma | ***E489**** | **Nonsense** | Diploid | 0.17 |
| TCGA-22-1016-01 | Lung Squamous Cell Carcinoma | ***V491L*** | **Missense** | Diploid | 0.44 |
| TCGA-39-5035-01 | Lung Squamous Cell Carcinoma | ***G147V*** | **Missense** | Diploid | 0.34 |
| TCGA-43-6770-01 | Lung Squamous Cell Carcinoma | ***X233_splice*** | **Splice** | **ShallowDel** | 0.09 |
| TCGA-AG-A002-01 | Rectal Adenocarcinoma | ***H195Y*** | **Missense** | Diploid | 0.84 |
| TCGA-EE-A29N-06 | Cutaneous Melanoma | ***H195Y*** | **Missense** | Diploid | 0.39 |
| TCGA-AP-A1E0-01 | Uterine Endometrioid Carcinoma | ***H195Y*** | **Missense** | Diploid | 0.61 |
| TCGA-33-4587-01 | Lung Squamous Cell Carcinoma | ***Y633Lfs*2*** | **FS ins** | **Gain** | 0.14 |
| TCGA-55-8205-01 | Lung Adenocarcinoma | ***T798P*** | **Missense** | **Gain** | 0.2 |
| TCGA-55-8506-01 | Lung Adenocarcinoma | ***V670L*** | **Missense** | Diploid | 0.15 |
| TCGA-56-7731-01 | Lung Squamous Cell Carcinoma | ***G395V*** | **Missense** | Diploid | 0.07 |
| TCGA-62-A46R-01 | Lung Adenocarcinoma | ***A99S*** | **Missense** | Diploid | 0.22 |
| TCGA-92-7341-01 | Lung Squamous Cell Carcinoma | ***W477R*** | **Missense** | **Gain** | 0.48 |
| TCGA-L3-A524-01 | Lung Squamous Cell Carcinoma | ***X195_splice*** | **Splice** | Diploid | 0.3 |
| TCGA-RD-A8NB-01 | Diffuse Type Stomach Adenocarcinoma. | ***R708Q*** | **Missense** | Diploid | 0.11 |
| TCGA-L5-A8NQ-01 | Esophageal Squamous Cell Carcinoma | ***S692F*** | **Missense** | Diploid | 0.14 |
| TCGA-VR-AA7B-01 | Esophageal Squamous Cell Carcinoma | ***E37K*** | **Missense** | **Gain** | 0.18 |
| TCGA-HF-A5NB-01 | Mucinous Stomach Adenocarcinoma | ***N660Ifs*3*** | **FS del** | Diploid | 0.51 |
| TCGA-VQ-A8P2-01 | Mucinous Stomach Adenocarcinoma | ***N338D*** | **Missense** | Diploid | 0.76 |
| TCGA-VQ-A8E7-01 | Tubular Stomach Adenocarcinoma | ***Y202H*** | **Missense** | Diploid | 0.7 |
| TCGA-06-5416-01 | Glioblastoma Multiforme | ***E182D*** | **Missense** | **ShallowDel** | 0.57 |
| TCGA-NA-A5I1-01 | Uterine Carcinosarcoma/Uterine... | ***E182D*** | **Missense** | Diploid | 0.45 |
| TCGA-AA-3947-01 | Mucinous Adenocarcinoma of the... | ***V364A*** | **Missense** | Diploid | 0.33 |
| TCGA-AA-3977-01 | Colon Adenocarcinoma | ***G337E*** | **Missense** | Diploid | 0.63 |
| TCGA-AA-3984-01 | Colon Adenocarcinoma | ***D609N*** | **Missense** | **Gain** | 0.18 |
| TCGA-AA-A010-01 | Colon Adenocarcinoma | ***L628F*** | **Missense** | Diploid | 0.61 |
| TCGA-AA-A01P-01 | Colon Adenocarcinoma | ***T324S*** | **Missense** | Diploid | 0.21 |
| TCGA-AA-A022-01 | Colon Adenocarcinoma | ***D494G*** | **Missense** | **Gain** | 0.06 |
| TCGA-AG-A002-01 | Rectal Adenocarcinoma | ***N394H*** | **Missense** | Diploid | 0.74 |
| TCGA-24-1564-01 | Serous Ovarian Cancer | ***L450P*** | **Missense** | **ShallowDel** | 0.13 |
| TCGA-FF-A7CW-01 | Diffuse Large B-Cell Lymphoma | ***R115Q*** | **Missense** | Diploid | 0.55 |
| TCGA-QK-A8Z8-01 | Head and Neck Squamous Cell Carcinoma | ***W473L*** | **Missense** | **ShallowDel** | 0.22 |
| TCGA-BA-A6DA-01 | Head and Neck Squamous Cell carcinoma | ***A296T*** | **Missense** | Diploid | 0.27 |
| TCGA-UF-A719-01 | Head and Neck Squamous Cell Ca... | ***L73S*** | **Missense** | **ShallowDel** | 0.55 |
| TCGA-CA-6717-01 | Mucinous Adenocarcinoma of the... | ***R514**** | **Nonsense** | Diploid | 0.45 |
| TCGA-EO-A22X-01 | Uterine Endometrioid Carcinoma | ***R514**** | **Nonsense** | Diploid | 0.43 |
| TCGA-D8-A1JG-01 | Breast Invasive Ductal Carcinoma | ***L162F*** | **Missense** | Diploid | 0.08 |
| TCGA-AX-A1CE-01 | Uterine Endometrioid Carcinoma | ***L162F*** | **Missense** | Diploid | 0.49 |
| TCGA-HZ-7922-01 | Pancreatic Adenocarcinoma | ***R716C*** | **Missense** | Diploid | 0.18 |
| TCGA-DK-A2I4-01 | Bladder Urothelial Carcinoma | ***G260**** | **Nonsense** | Diploid | 0.1 |
| TCGA-EE-A3AG-06 | Cutaneous Melanoma | ***P138S*** | **Missense** | **Gain** | 0.96 |
| TCGA-EE-A2MR-06 | Cutaneous Melanoma | ***S44L*** | **Missense** | Diploid | 0.19 |
| TCGA-ER-A1A1-06 | Cutaneous Melanoma | ***P780S*** | **Missense** | Diploid | 0.11 |
| TCGA-06-5416-01 | Glioblastoma Multiforme | ***D427N*** | **Missense** | **ShallowDel** | 0.25 |
| TCGA-FI-A2D5-01 | Uterine Endometrioid Carcinoma | ***D427N*** | **Missense** | Diploid | 0.41 |
| TCGA-DD-AAW2-01 | Hepatocellular Carcinoma | ***X195_splice*** | **Splice** | **Gain** | 0.9 |
| TCGA-EE-A183-06 | Cutaneous Melanoma | ***S280Y*** | **Missense** | Diploid | 0.11 |
| TCGA-AX-A05Z-01 | Uterine Endometrioid Carcinoma | ***S280Y*** | **Missense** | Diploid | 0.06 |
| TCGA-ND-A4WC-01 | Uterine Carcinosarcoma/Uterine... | ***D597E*** | **Missense** | Diploid | 0.33 |
| TCGA-ZF-A9RG-01 | Bladder Urothelial Carcinoma | ***L116F*** | **Missense** | Diploid | 0.22 |
| TCGA-GN-A26C-01 | Cutaneous Melanoma | ***S409L*** | **Missense** |  | 0.32 |
| TCGA-DU-6392-01 | Astrocytoma | ***A25V*** | **Missense** | Diploid | 0.38 |
| TCGA-GC-A6I1-01 | Bladder Urothelial Carcinoma | ***X666_splice*** | **Splice** | **Gain** | 0.16 |
| TCGA-LL-A8F5-01 | Breast Invasive Ductal Carcinoma | ***S545**** | **Nonsense** | **Gain** | 0.14 |
| TCGA-EK-A2RN-01 | Cervical Squamous Cell Carcinoma | ***I618M*** | **Missense** | Diploid | 0.08 |
| TCGA-VS-A958-01 | Cervical Squamous Cell Carcinoma | ***T803I*** | **Missense** | Diploid | 0.21 |
| TCGA-ZJ-AB0H-01 | Cervical Squamous Cell Carcinoma | ***T496A*** | **Missense** | Diploid | 0.03 |
| TCGA-AA-A00N-01 | Mucinous Adenocarcinoma of the... | ***K625T*** | **Missense** | Diploid | 0.1 |
| TCGA-AU-6004-01 | Colon Adenocarcinoma | ***Y613H*** | **Missense** | Diploid | 0.11 |
| TCGA-CM-6171-01 | Colon Adenocarcinoma | ***G272Vfs*10*** | **FS del** | Diploid | 0.3 |
| TCGA-AZ-6605-01 | Colon Adenocarcinoma | ***ACE2-GPR143*** | **Fusion** | **ShallowDel** |  |
| TCGA-06-1801-01 | Glioblastoma Multiforme | ***I358F*** | **Missense** | Diploid | 0.56 |
| TCGA-06-5416-01 | Glioblastoma Multiforme | ***P178S*** | **Missense** | **ShallowDel** | 0.3 |
| TCGA-4A-A93Y-01 | Papillary Renal Cell Carcinoma | ***F762L*** | **Missense** | Diploid | 0.03 |
| TCGA-DU-6392-01 | Astrocytoma | ***I679N*** | **Missense** | Diploid | 0.4 |
| TCGA-DU-6392-01 | Astrocytoma | ***A396T*** | **Missense** | Diploid | 0.36 |
| TCGA-VG-A8LO-01 | Serous Ovarian Cancer | ***PIR-ACE2*** | **Fusion** | **Gain** |  |
| TCGA-2J-AABP-01 | Pancreatic Adenocarcinoma | ***V293I*** | **Missense** | Diploid | 0.04 |
| TCGA-D3-A2JB-06 | Cutaneous Melanoma | ***G272C*** | **Missense** | Diploid | 0.14 |
| TCGA-D3-A2JP-06 | Cutaneous Melanoma | ***W610L*** | **Missense** | Diploid | 0.09 |
| TCGA-D3-A2JP-06 | Cutaneous Melanoma | ***G268C*** | **Missense** | Diploid | 0.11 |
| TCGA-D3-A2JP-06 | Cutaneous Melanoma | ***Q18K*** | **Missense** | Diploid | 0.09 |
| TCGA-D3-A8GI-06 | Cutaneous Melanoma | ***A311V*** | **Missense** | Diploid | 0.84 |
| TCGA-EE-A183-06 | Cutaneous Melanoma | ***Q598H*** | **Missense** | Diploid | 0.09 |
| TCGA-EE-A20F-06 | Cutaneous Melanoma | ***M383I*** | **Missense** | Diploid | 0.08 |
| TCGA-EE-A2GT-06 | Cutaneous Melanoma | ***E312**** | **Nonsense** | Diploid | 0.1 |
| TCGA-ER-A19H-06 | Cutaneous Melanoma | ***E22D*** | **Missense** | Diploid | 0.1 |
| TCGA-FR-A729-06 | Cutaneous Melanoma | ***S317F*** | **Missense** | **Gain** | 0.39 |
| TCGA-Z2-A8RT-06 | Cutaneous Melanoma | ***E489K*** | **Missense** | **ShallowDel** | 0.44 |
| TCGA-A5-A0G1-01 | Uterine Serous Carcinoma/Uteri... | ***D269Y*** | **Missense** | Diploid | 0.22 |
| TCGA-A5-A0GX-01 | Uterine Endometrioid Carcinoma | ***Y454**** | **Nonsense** | Diploid | 0.1 |
| TCGA-A5-A1OF-01 | Uterine Mixed Endometrial Carc... | ***R518M*** | **Missense** | Diploid | 0.24 |
| TCGA-A5-A2K2-01 | Uterine Serous Carcinoma/Uteri... | ***X268_splice*** | **Splice** | **ShallowDel** | 0.55 |
| TCGA-AJ-A3BH-01 | Uterine Endometrioid Carcinoma | ***L760M*** | **Missense** | Diploid | 0.18 |
| TCGA-AP-A1DK-01 | Uterine Endometrioid Carcinoma | ***W48L*** | **Missense** | Diploid | 0.25 |
| TCGA-AP-A1DV-01 | Uterine Endometrioid Carcinoma | ***N437H*** | **Missense** | Diploid | 0.42 |
| TCGA-B5-A1MR-01 | Uterine Endometrioid Carcinoma | ***V658L*** | **Missense** | Diploid | 0.14 |
| TCGA-B5-A1MR-01 | Uterine Endometrioid Carcinoma | ***L120I*** | **Missense** | Diploid | 0.17 |
| TCGA-B5-A3FA-01 | Uterine Endometrioid Carcinoma | ***W477Vfs*18*** | **FS ins** | Diploid | 0.12 |
| TCGA-B5-A3FA-01 | Uterine Endometrioid Carcinoma | ***E35K*** | **Missense** | Diploid | 0.43 |
| TCGA-B5-A5OD-01 | Uterine Serous Carcinoma/Uteri... | ***K481=*** | **Splice** | **Gain** | 0.15 |
| TCGA-BS-A0UV-01 | Uterine Endometrioid Carcinoma | ***M82T*** | **Missense** | Diploid | 0.09 |
| TCGA-EO-A22R-01 | Uterine Endometrioid Carcinoma | ***N578S*** | **Missense** | Diploid | 0.35 |
| TCGA-EO-A22R-01 | Uterine Endometrioid Carcinoma | ***Y497C*** | **Missense** | Diploid | 0.35 |
| TCGA-EO-A22U-01 | Uterine Endometrioid Carcinoma | ***E571G*** | **Missense** | Diploid | 0.42 |
| TCGA-EO-A22U-01 | Uterine Endometrioid Carcinoma | ***R393I*** | **Missense** | Diploid | 0.49 |
| TCGA-EO-A3B0-01 | Uterine Endometrioid Carcinoma | ***F72C*** | **Missense** | Diploid | 0.37 |
| TCGA-EY-A5W2-01 | Uterine Endometrioid Carcinoma | ***R115W*** | **Missense** | Diploid | 0.49 |
| TCGA-FI-A2D0-01 | Uterine Endometrioid Carcinoma | ***D201Ifs*9*** | **FS del** | Diploid | 0.25 |
| TCGA-FI-A2D5-01 | Uterine Endometrioid Carcinoma | ***A576T*** | **Missense** | Diploid | 0.37 |
