# Supplementary material for: Genetic alteration, RNA expression, and DNA methylation profiling of coronavirus disease 2019 (COVID-19) receptor ACE2 in malignancies: a pan-cancer analysis
Source: J Hematol Oncol. 2020 May 4;13:43. doi: 10.1186/s13045-020-00883-5 (PMC7197362; doi:10.1186/s13045-020-00883-5)
Supplement: Supplementary file 3 — Additional file 3: Table S2. The TPM expression of ACE2 in 30 kinds of tumors from TCGA database. [file 13045_2020_883_MOESM3_ESM.docx]

| Kinds | Tumor | Normal |
| --- | --- | --- |
| ACC | 1.03 | 0.45 |
| BLCA | 0.52 | 0.59 |
| BRCA | 0.1 | 0.88 |
| CESC | 1.32 | 0.33 |
| CHOL | 1.96 | 1.13 |
| COAD | 11.35 | 1.06 |
| DLBC | 0.01 | 0 |
| ESCA | 1.39 | 1.7 |
| GBM | 0.09 | 0.08 |
| HNSC | 1.38 | 1.46 |
| KICH | 0.09 | 5.01 |
| KIRC | 24.04 | 12.24 |
| KIRP | 39.08 | 11.06 |
| LAML | 0 | 0.03 |
| LGG | 0.11 | 0.08 |
| LIHC | 0.33 | 0.57 |
| LUAD | 1.71 | 0.85 |
| LUSC | 1.19 | 0.83 |
| OV | 0.21 | 0.92 |
| PAAD | 3.03 | 0.96 |
| PCPG | 0.09 | 0.93 |
| PRAD | 0.16 | 0.41 |
| READ | 13.42 | 0.76 |
| SARC | 0.07 | 37.23 |
| SKCM | 0.05 | 0.31 |
| STAD | 1.82 | 0.32 |
| TGCT | 0.35 | 34.67 |
| THCA | 0.82 | 2.8 |
| THYM | 0.38 | 0 |
| UCEC | 0.51 | 0.41 |
| UCS | 0.13 | 0.39 |

**Table S2: The TPM expression of ACE2 in 30 kinds of tumors from TCGA database.**
