# Supplementary material for: Genetic alteration, RNA expression, and DNA methylation profiling of coronavirus disease 2019 (COVID-19) receptor ACE2 in malignancies: a pan-cancer analysis
Source: J Hematol Oncol. 2020 May 4;13:43. doi: 10.1186/s13045-020-00883-5 (PMC7197362; doi:10.1186/s13045-020-00883-5)
Supplement: Supplementary file 4 — Additional file 4: Table S3. Dataset sources used in the study. [file 13045_2020_883_MOESM4_ESM.docx]

| **Dataset Sources** | **Assay** | **Location** | **Website** |
| --- | --- | --- | --- |
| cBioPortal | Genetic alterations (Cancer type Summary) | Figure S1A, S1B | https://www.cbioportal.org |
|  | Mutation annomination & structural prediction | Figure S2A, S2B, S2C,S2D, Table S1 |  |
|  | RNA-seq of Mutation & copy number variation | Figure S3A, S3B |  |
| Gepia2 | Expression profiling | Figure 1A, 1B, 1C, Figure S4, Table S2 | http://gepia2.cancer-pku.cn/ |
|  | Survival Analysis | Figure S7, S8, S9 |  |
| Ualcan | DNA methylation assay | Figure 3, Figure S5, S6 | http://ualcan.path.uab.edu |
|  | Expression profiling | Figure 1A |  |

**Table S3. Dataset sources used in the study**
